# A Novel Secure IoT-Based Smart Home Automation System Using a Wireless Sensor Network

**DOI:** 10.3390/s17010069

**Published:** 2016-12-30

**Authors:** Sandeep Pirbhulal, Heye Zhang, Md Eshrat E Alahi, Hemant Ghayvat, Subhas Chandra Mukhopadhyay, Yuan-Ting Zhang, Wanqing Wu

**Affiliations:** 1 Institute of Biomedical and Health Engineering, Shenzhen Institutes of Advanced Technology, Shenzhen 518055, China; sandeep@siat.ac.cn (S.P.); hy.zhang@siat.ac.cn (H.Z.); 2 Research Center for Biomedical Information Technology, Shenzhen Institutes of Advanced Technology, 1068 Xueyuan Avenue, Shenzhen University Town, Shenzhen 518055, China; 3 Shenzhen College of Advanced Technology, University of Chinese Academy of Sciences, Shenzhen 518055, China; 4 School of Engineering and Advanced Technology, Massey University, Palmerston North 4442, New Zealand; ealahi@yahoo.com (M.E.E.A.); ghayvat@gmail.com (H.G.); 5 Department of Engineering, Macquarie University, Sydney 2109, Australia; 6 Joint Research Centre for Biomedical Engineering, Chinese University of Hong Kong, Shatin N.T., Hong Kong, China; ytzhangapple@icloud.com

**Keywords:** energy-efficient, home automation, internet of things, security, wireless sensor networks

## Abstract

Wireless sensor networks (WSNs) provide noteworthy benefits over traditional approaches for several applications, including smart homes, healthcare, environmental monitoring, and homeland security. WSNs are integrated with the Internet Protocol (IP) to develop the Internet of Things (IoT) for connecting everyday life objects to the internet. Hence, major challenges of WSNs include: (i) how to efficiently utilize small size and low-power nodes to implement security during data transmission among several sensor nodes; (ii) how to resolve security issues associated with the harsh and complex environmental conditions during data transmission over a long coverage range. In this study, a secure IoT-based smart home automation system was developed. To facilitate energy-efficient data encryption, a method namely Triangle Based Security Algorithm (TBSA) based on efficient key generation mechanism was proposed. The proposed TBSA in integration of the low power Wi-Fi were included in WSNs with the Internet to develop a novel IoT-based smart home which could provide secure data transmission among several associated sensor nodes in the network over a long converge range. The developed IoT based system has outstanding performance by fulfilling all the necessary security requirements. The experimental results showed that the proposed TBSA algorithm consumed less energy in comparison with some existing methods.

## 1. Introduction

In 21th century, the Internet of Things (IoT) is one of the most constructive and dominant wireless communication models. It is the common conception of things that are identifiable, readable, controllable, addressable, and locatable via the Internet. In the IoT surroundings, all entities of our everyday life can be associated with the Internet owing to their computing and communication capabilities. IoT enlarges the perception of the Internet and makes it more suitable for different applications. Due to this reason, IoT has become more beneficial in numerous domains such as health monitoring, assisted living monitoring, and smart home automation systems [1]. In these systems, several kinds of sensors are connected wirelessly to develop distributed networks. The wireless sensor networks (WSNs) is one of the most essential technologies utilized in IoT-based smart home automation. These are distributed networks of tiny and light weighted wireless sensor nodes, which could be extended depending on the requirement of physical parameters such as pressure, temperature, and relative humidity. Each sensor node in the WSNs includes three subsystems i.e., sensor subsystem for environment sensing, processing subsystem for computation of the sensed information, and a communication subsystem for exchanging the collected data between different sensors [1,2,3,4,5,6]. Several studies have integrated WSNs with Internet Protocol (IP) to develop IoT for offering real-time connectivity of all devices from everyday life at anytime and anywhere [7]. In the IoT based applications, WSNs are considered as the most significant components which collect the real-time sensed data from their surrounding environment [8,9]. The traditional WSNs offers a closed system designed for specific applications, however, IoT-based applications are focused to develop a large-scale WSNs infrastructure which could support open standard and are independent of specific applications [10].

In most of the IoT-based home automation systems, the actuators and sensors are positioned within the home environment to control and supervise its operations. Afterward, these devices are connected to the local server via a wireless medium for data collection and analysis. One of the most important issues is how to securely transmit the collected data from the sensor nodes to the appropriate destination. Therefore, several mechanisms have been proposed to solve this challenging issue including traditional encryption techniques and security methods developed for WSNs [11,12,13,14]. Sensor nodes in these networks have limited resources including restricted power supply, memory, limited data processing capability, and range of communication [15,16]. Another major issue is how to efficiently utilize these limited resources for several IoT based applications. Hence, security algorithms which could consume less energy for data encryption should be utilized in order to make efficient use of available resources in their networks. Additionally, in IoT based smart home, a large number of devices are connected to the internet at a long distance. Third most significant issue is how to increase coverage range to provide a communication framework combining the sensor and backhaul networks [17]. Hence, highly secured IoT based smart home systems with could provide a balance between level of security, energy-efficient security algorithm implementation based on efficient key generation mechanism for data encryption, and capability of network to support communication among large number of IoT nodes at wide coverage range is much needed.

In this study, a secured IoT based home automation platform was developed. The contribution of this paper is in threefold; first, the development of novel IoT based hardware platform by using Intel Galileo Board, TM936 sensor and N-2230 Intel Centrino Wi-Fi module for the collection of environmental temperature. Second, an energy-efficient security algorithm namely Triangle Based Security Algorithm (TBSA) on basis of simple and efficient key generation mechanism was proposed for data encryption. Third, the performance of proposed TBSA is compared with traditional encryption ciphers and security methods designed for WSNs in terms of energy efficiency.

This research paper is organized as follows: Section 2 comprises of related works. In Section 3, security requirements in IoT based home automation using WSNs are discussed. Section 4 presents the methods and implementation while the experimental results and discussion are described in Section 5. The paper is concluded in Section 6.

## 2. Related Work

Recently, IoT has been applied in numerous applications including smart home monitoring systems for assisted living to predict the wellness of residents through the monitoring of several home appliances [4], building management framework to support energy-saving applications [18], and human activity patterns monitoring [19,20] among others. As the Internet communications infrastructure develops to include sensing objects, suitable mechanisms are needed to secure communications with such entities, in the context of IoT applications. In real world IoT applications, security threats and attacks are becoming a major issue with respect to data transmission. Hence, it is extremely crucial that the IoT based system should include security mechanism that could resist possible security threats and attacks in the form of data modification, impersonation, and eavesdropping, among others. According to [21], IEEE 802.15.4 standard based IPv6 over Low power Wireless Personal Area Networks (6LoWPAN) in the integration of WSNs with internet is used for providing secure data transmission in IoT. However, despite of fact that 6LoWPAN-based IoT systems require less energy for implementing security, it is still not appropriate for smart homes due to two major issues: (1) An efficient key generation mechanism is not included in IEEE 802.15.4 sandard so how to securely add a new device into the network or to manage the cryptographic keys is also not explained in that standard [22]. One possible solution for efficient key generation and management in 6LoWPAN is Lightweight IKEv2, but it requires more resources and energy for its implementation [23,24]; (2) It is not an appropriate standard for smart homes, since it does not facilitate communication among a large number of IoT nodes and it also does not have a wide coverage range [17]. The first issue can be resolved by developing an energy-efficient security algorithm based on an efficient key generation mechanism for secure data transmission in IoT applications. To resolve the second problem, recently low-power Wi-Fi systems optimized for sensing applications are available due to the growing industry requirements for smart objects having IP connectivity [25]. According to [17], the latest Wi-Fi standard fills this gap by combining the advantages of Wi-Fi and low-power sensor network communication technologies. The emerging Wi-Fi standard is a promising communication standard that supports a massive number of heterogeneous devices in the IoT. A comparison between the latest 802.11 ah and 802.15.4 standards is described in detail in [17]; 802.11 ah performs better in terms of association time, throughput, delay, and coverage range. Due to all these advantages, Wi-Fi is the preferred standard over 6LoWPAN for several IoT applications such as smart cities and smart homes. Therefore, along with the low power Wi-Fi module to support large number of IoT nodes and to increase coverage range, security algorithm for data encryption based on efficient key generation mechanism need to be included in WSNs with internet to develop novel secured IoT based smart home.

Several IoT based systems are developed which includes integration of security mechanisms within WSNs to provide efficient security for different applications [26,27,28]. Generally, hash functions, symmetric and asymmetric encryption algorithms are utilized to offer data security. The asymmetric algorithms are not suitable for implementing security in sensor networks, due to the limited computational power of the tiny sensor nodes [6]. Thus, hash functions, symmetric algorithms including message digest 4 (MD4) [29], message digest 5 (MD5) [30], secure hash algorithm 1 (SHA-1) [31], hash message authentication code (HMAC) [32], Data Encryption Standard (DES) [33], Advanced Encryption Standard (AES) [34], Rivest Cipher 4 (RC4) [35], blowfish [36], are utilized to secure the sensor networks. Since, these mentioned techniques are not precisely developed by keeping in view the specification of WSNs; so these networks require more energy for their implementation. Therefore, security mechanisms specifically designed for WSNs could be the optimal solution for IoT applications. Mandal et al. developed a hybrid scheme of both symmetric-key and asymmetric-key based cryptographic functions for securing WSNs. However, their scheme has not considered all the major security requirements [16]. Aakash et al. proposed a novel hybrid lightweight security method namely; PRESENT-GRP for secure data transmission in IoT based applications and it was implemented on an Intel Galileo Gen 2 board. It follows a complex permutation boxes-based strategy, which requires more processing time and resources [26]. Wood et al. proposed an alarm-net system to monitor residential and assisted-living by query protocols. Their system is vulnerable to adversarial data confidentiality attacks which may reveal the location of the resident, and also it requires several resources that need more execution time for data encryption [27]. Mian et al. designed a lightweight payload based mutual authentication method namely; PAWN for cluster based hierarchical WSNs. Their proposed method is based on two steps: firstly, an election of cluster heads for coordinating the neighboring nodes in the network. Secondly, each cluster head acts as server for valid authentication of the nodes to initiate data transmission. The major drawback of their approach is that more resources are required to accomplish the cluster heads election procedure [37].

The challenging problem with all of the aforementioned traditional security mechanisms designed for WSNs in providing data security methods is that they utilize complex strategies in their respective system, so more resources are required. Therefore, in this research security algorithm based on the simple and efficient key generation mechanism is proposed namely; TBSA, it consumes less processing time and energy for data encryption. The low-power Wi-Fi optimized for sensing applications has advantages of better association time, throughput, delay, and coverage range; therefore it is preferred in this research. This study develops a IoT based home automation system by integrating the low power Wi-Fi and proposed TBSA in WSNs with internet, it increases the coverage range and has the capability to support a large number of IoT devices. Additionally, the proposed IoT system accomplishes all the necessary security requirements including confidentiality, privacy, integrity, data freshness, secure localization, non-repudiation, availability, access control, trustworthiness, and authentication for protecting the network from critical security attacks and threats.

## 3. Security Requirements in IoT Based Home Automation Using WSNs

Security is one of the most imperative aspects of any system. People have distinct perceptions concerning security and therefore it is defined in numerous ways. Generally, security helps protect the entire system from external as well as internal risks. At present, WSNs are useful platforms in IoT-based smart home applications, and data transmission in these networks is usually done through wireless medium. This could cause several severe attacks to the networks as well as pose security threats to WSNs. This section describes the main requirements for implementing security in IoT based applications [38,39,40].

### 3.1. Authentication

Authentication is one of the requirements in any IoT-based application; it usually deals with the impersonating threats. In IoT-based automation systems using WSNs, all the sensor nodes transmit data to the server by the wireless medium. An attacker can effortlessly insert messages into the system, thus the destination node needs to confirm that the information utilized in any decision making procedure is initiated from the proper source node. Basically, authentication permits the destination node to verify if the information was transmitted from the appropriate source node.

### 3.2. Trustworthiness

It is the ability of a system to authenticate the identity and ascertain trust in a third party. Third-party trust is a situation in which the source and destination nodes in IoT-based application can implicitly trust each other although they have not established communication paths for data transmission beforehand.

### 3.3. Data Freshness

An attacker can capture the information during its transmission from source to destination and replay it afterward by utilizing older keys to puzzle the coordinator. Data freshness implies that the information is fresh and nobody can replay old information.

### 3.4. Confidentiality and Privacy

In WSNs, it is required to defend the original information from any disclosure. A WSN should not disclose the original information from source node to the neighboring or even external networks. In IoT-based smart home applications, the sensor nodes accumulate and forwards specific information to the server. An attacker can eavesdrop on the data transmission, and can overhear significant data. This eavesdropping phenomenon can lead to severe damage since the adversary can utilize the captured information for numerous illegal purposes. Therefore, confidentiality ensures that only verified users can receive the information. Along with that, privacy is also an important concern to guarantee that all sensor nodes in the system fulfill the privacy policies and help them to manage their specific data.

### 3.5. Secure Localization

Most WSN-based applications necessitate an exact assessment of the source location. Lack of intelligent tracking procedures can permit the attacker to transmit incorrect information about the source location by stating fake signal strengths. The secured localization is very important for tracking the actual source node for data transmission.

### 3.6. Integrity

Beside confidentiality and privacy, integrity is also an important security factor during the transmission of data in WSNs. An attacker can always change the information by inserting some fragments of fake information within the transmitted message to alter the originally meaning. This altered data can be sent to the destination node. Therefore, an integrity mechanism is very significant to protect the original data from external attacks.

### 3.7. Non-Repudiation

It is the ability of a system to validate occurrence or non-occurrence of an action from the source nodes. In IoT-based smart home automation, it is important to ensure that the source nodes should not deny their authenticity when sending the messages that are originated from them.

### 3.8. Availability

This property allows reliable access of system resource in timely manner to valid sensor nodes in the network. In IoT-based applications, it is very essential that network resources should be available to the appropriate nodes.

### 3.9. Access Control

To keep out potential attackers, it is needed to recognize each user and each device so as to enforce security policies. Therefore, noncompliant sensor nodes within the network need to be blocked or given only limited access. This process is known as network access control (NAC). To develop a secured IoT-based system, it is extremely crucial that the system should fulfill all the above mentioned security requirements that could oppose different security attacks like replaying, data modification, impersonation, and eavesdropping among others.

## 4. Method and Implementation

### 4.1. Secured IoT-Based System

This research aimed to develop a secure IoT-based system using WSNs in which collected data can be securely and efficiently transmitted between source and destination nodes. The specific information from each sensor is also stored on the server, and the data can be shared with the proper destination upon validation. Therefore, secure data transmission is necessary for home automation-based applications. All security constraints (stated in Section 3) are divided into two categories: network security and data security. Network security requirements include secured localization, non-repudiation, availability, access control, trustworthiness and authentication while data security requirements are confidentiality, privacy, integrity, and data freshness. This research fulfills network security requirements by developing a secured IoT-based platform driven by the proposed TBSA to accomplish data security constraints.

**Figure 1:**
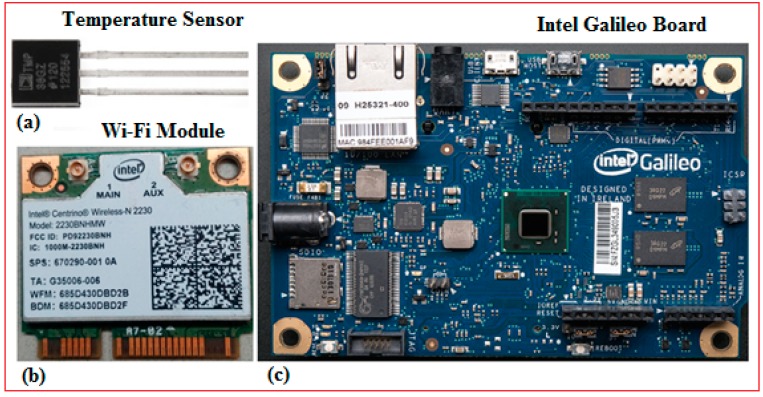
The components for the sensor node: (**a**) temperature Sensor; (**b**) Wi-Fi Module (N-2320); (**c**) Intel Galileo Board.

In this study, WSN was developed at School of Engineering and Advanced Technology (SEAT), Massey University, Palmerston North, New Zealand. The TMP36 temperature sensors were used for capturing environmental temperature which served as the dataset in the study. The components used to build the sensor nodes are shown in Figure 1. The temperature sensor is presented in Figure 1a, and Figure 1b shows the Wi-Fi module (Intel Centrino Wireless N-2230) which were both integrated on an Intel Galileo based microcontroller board as demonstrated in Figure 1c. The Galileo board includes a 32-bit Intel Pentium-class system mounted on a chip that contains an Arduino Software Development Environment (IDE), together with an Intel processor for fast processing of data. This board is used to develop an intelligent wireless sensor node. Also, the Wi-Fi module was mounted on the board alongside the temperature sensor to produce a sensor node. In smart home-based applications, it is very important that sensor nodes be updated with information received from the remote server located at an extensive distance with good speed and reliability. Therefore, Intel Centrino Wireless N-2230 was utilized in our research for transmitting the information over a wide coverage range. This module enhances reliability and speeds up to 300 Mbps for data transmission. 

**Figure 2:**
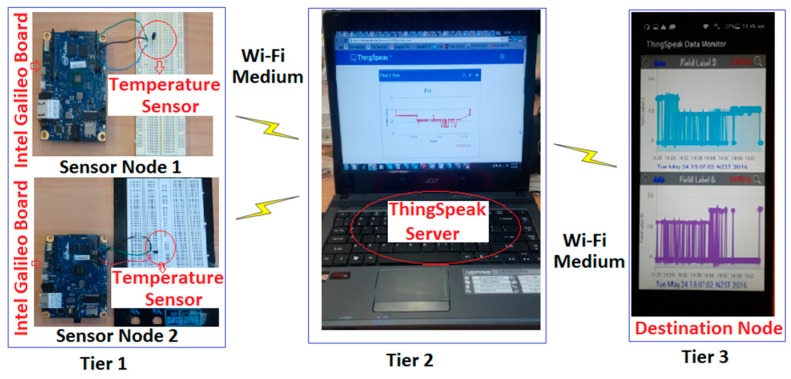
The hardware implementation of WSNs.

The architecture of WSNs including three tiers for data transmission between the source and destination nodes is shown in Figure 2. In tier 1, the sensor nodes were designed to collect data by using Wi-Fi as a wireless medium, the sensed data is then stored on the server in tier 2. In this study, a ThingSpeak server was used to store the recorded data. Finally in tier 3, the stored data was transmitted to the destination node after proper authentication. 

**Figure 3:**
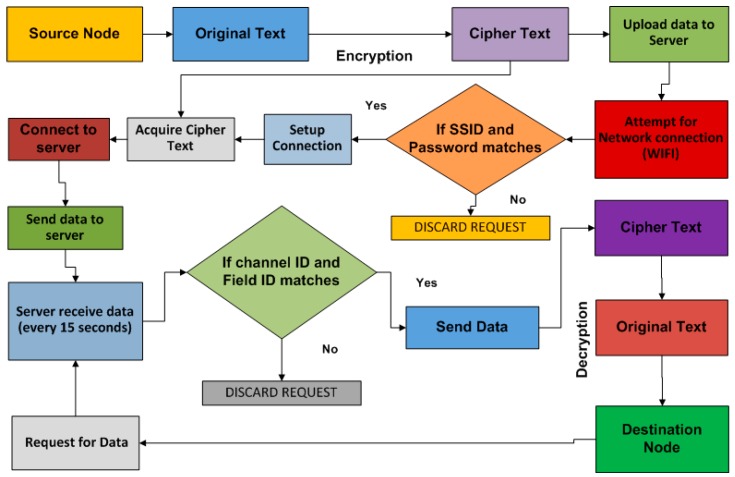
Source-destination data communication model.

The communication model between the source and destination nodes is shown in Figure 3. The source node generates information which is encrypted by using an encryption algorithm. It is very important to convert original text into cipher text, so it can be interpreted by proper destination. Different encryption algorithms including MD4, MD5, SHA-1, HMAC, DES, AES, RC4, blowfish, security algorithms for WSNs, and the proposed TBSA were examined in this study. It was observed from the conducted experiments that the compared existing encryption algorithms consumed more energy than the proposed TBSA, because they require more overheads and complex procedures to encrypt the original information. The TBSA algorithm was specially developed for all applications which involve the transmission of information among wireless sensor nodes. Since the sensor nodes in WSNs have problem of limited resources such as memory, restricted power supply, and data processing power, the TBSA encryption method is used as a possible solution to achieve energy-efficient security for IoT-based applications.

In our IoT-based system, when a sensor node needs to transmit the periodical information to the server, then the server needs to verify the identity of the source node. The next step is the anonymous confirmation phase where the communication to be initiated from the source node to server is validated. A unique key (K_API_) is assigned to each sensor node for accessing the channel of the server through the secured medium. The communication can be initiated on the basis of K_API_ confirmation in order to develop proper trust between the source and destination nodes. After this, the server produces a tracking sequence (*Ts*), which is a sequence value of 32-bit. This sequence value was arbitrarily generated on the basis of observing the location of the source node. In particular, for each request of the sensor node, the server produces arbitrary value *n* and then locates *Ts* = *n* and maintains a record in its database, which can assist the server to observe and record the most current *Ts* for each unique K_API_ of every sensor node in the network. This sequence value can be utilized to accelerate the authentication procedure as well as to avoid any replay endeavor from any attacker, and by looking at the *Ts* value in comparison with the accumulated number in the database, the server can figure out the source node. Now, source nodes cannot deny their authenticity of the message sent from them. Now, during the execution of the anonymous authentication phase, if the *Ts* provided by any sensor node do not match with the accumulated value in the server then the server instantly ends the connection. In the case where the server cannot locate any *Ts*, the server will need to validate the reserved key (K_ID_) for emergency data transmission, thereafter it attempts to identify the tracking sequence number for emergency data (*Te*). If successfully validated, then data transmission between the source node and the server can be started. Furthermore, the next level of authentication for the network involves service set identifier (SSID) and network password validation. Firstly, at this level of authentication the SSID and network password validation will be checked. After proper validation of the SSID and password, the server then checks the channel and field IDs. The cipher text generated from the source node using the proposed TBSA is transmitted to the server if there is match between IDs of the source node and the server. To keep out potential attackers, it is needed to recognize each user and each device in order to enforce security policies. After proper access control, all network resources will be available in timely manner to the appropriate sensor nodes. This study considered the ThingSpeak server to store the information generated from every sensor node and the collected data are being updated to the server after every 15 s. The HTTP/1.1 protocol of the application layer is used to send the cipher text directly to the server. The request generated by the destination node to acquire information will be fulfilled if unique channel ID and field ID matches. Thereafter, the destination node would be able to decrypt the cipher text by using a specific authentication key.

### 4.2. Proposed TBSA Security Method

**Figure 4:**
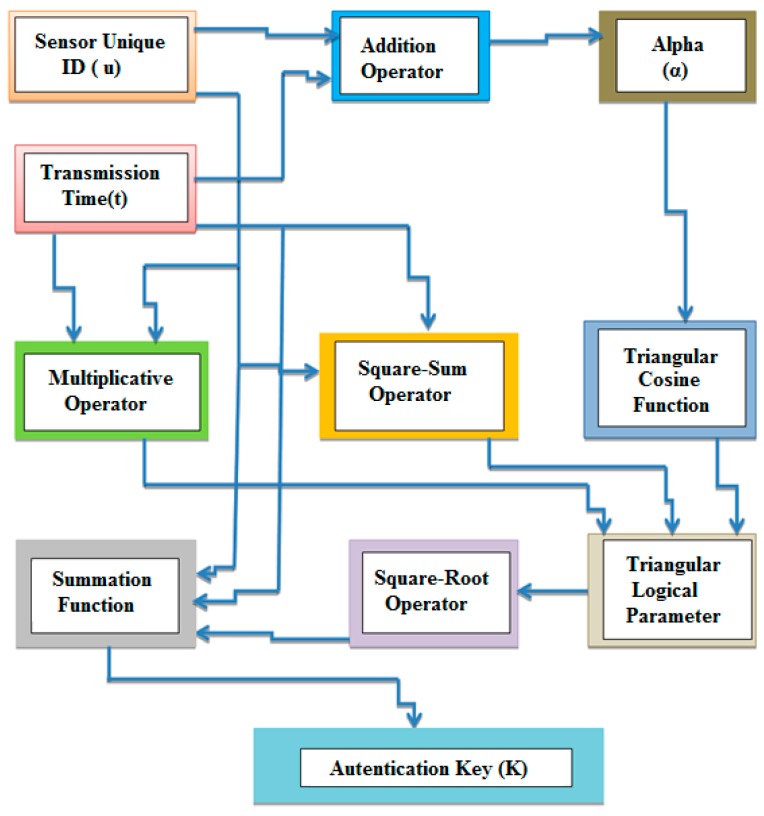
Key generation mechanism for proposed TBSA.

Data security is of the major concerns in a smart home automation infrastructure. It is necessary to ensure that the server receives actual information from specific source nodes. Therefore, encryption schemes are often utilized to guarantee data security in WSNs. In this study, we proposed the TBSA method in order to attain confidentiality, privacy, integrity, and the data freshness with a realistic computational overhead. Our proposed TBSA method is simple because it eradicates the utilization of complex key generation procedures. TBSA is compatible with prompt and secure data transmission, the encryption technique can ensure both the integrity and secrecy of the information without any supplementary cryptographic primitive, like the CRC support, MAC, and hash functions. However, in WSNs, those techniques, which are based on complicated key generation mechanisms, necessitate high computational cost for the management of keys and as well consume a lot of time and energy during data transmission. This research aims to produce resource efficient security algorithm, which could provide energy-efficient security for home automation based applications. The proposed TBSA security algorithm is based on the non-right angle triangle key generation procedure. The block diagram of the efficient key generation mechanism for proposed TBSA is shown in Figure 4. The authentication key (K) generated from proposed key generation procedure is used to provide unique authentication for data transmission between the source and destination nodes.

Consider a sensor node that transmits the specific data collected form TMP36 at time (t) by using the proposed TBSA, and sensor’s unique identification (ID) is represented as (*u*). Furthermore, t and *u* are given as input to the square-sum and multiplicative operators. The multiplicative operator simply multiplies the t and u, and the outcome of this operator is denoted by *m* as shown in Equation (1). The square-sum operator initially squares the *t* and *u* values independently and then the squared values are summed to produce the final output (*A*) as shown in Equation (2):

(1) m=(u)*(t)

(2) $$A=t^{2}+u^{2}$$

**Figure 5:**
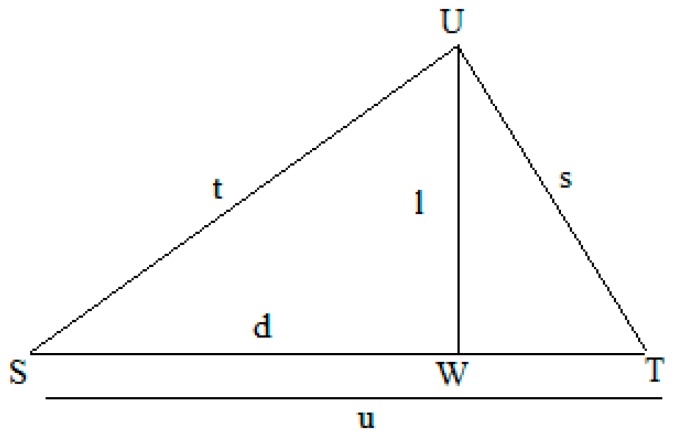
Triangle STU.

The triangular logical function is further used to produce logical parameter, the input to this function includes three values such as *t*, *u*, and *α*. Consider a *STU* triangle, which does not include a right angle. The *t* and *u* are acting as two sides of the *STU* triangle as shown in Figure 5, where addition of these two values is acting as the corresponding angle (α) for the third side (s) of triangle *STU* as shown in Equation (3), and the line *UW* drawn from the vertex *U* is perpendicular to *ST*. Now, *SUW* is a right angle triangle, *l* and *d* are its two sides, the values of these sides can be calculated by Equations (4) and (5). Furthermore, *l*, *u* and *d* are used to calculate third side (s) of triangle *STU* by applying Pythagoreans theorem as shown in Equation (6). By substituting values of *l* and *d* in Equation (6), we get Equation (7). By expanding the right hand side of Equation (7), we obtained Equations (8) and (9):

(3) α=(t+u)/2

(4) sin∠ α=l/t⇒l=t sin∠ α

(5) cos∠ α=d/t⇒d=t cos∠ α

By applying Pythagorean Theorem in triangle *STU*, we have:

(6) $$s^{2}=l^{2}+{\left(u-d\right)}^{2}$$

By substituting for *d* and *l* we have:

(7) $$s^{2}={\left(t\;\text{sin}∠\;\alpha \right)}^{2}+{\left(u-(t\;\text{cos}∠\;\alpha )\right)}^{2}$$

(8) $$s^{2}=t^{2}\;{\text{sin}}^{2}∠\alpha +u^{2}-2ut\;\text{cos}∠\alpha +t^{2}\;{\text{cos}}^{2}∠\alpha$$

(9) $$s^{2}=t^{2}({\text{sin}}^{2}∠\alpha +{\text{cos}}^{2}∠\alpha )+u^{2}-2tu\;\text{cos}∠\alpha$$

As ${\text{sin}}^{2}∠\;\alpha +{\text{cos}}^{2}∠\;\alpha =1$, by putting this value in Equation (9), we get Equation (10):

(10) $$s^{2}=t^{2}(1)+u^{2}-2tu\;\text{cos}∠\alpha$$

Triangular cosine function is applied to the angle (α) as demonstrated in Equation (11):

(11) alpha=cos(α)

By inserting the output values from Equations (1), (2) and (11) into Equation (10) to generate triangular logical parameter (*s*^2^) as shown in Equation (12):

(12) $$s^{2}=A-2m*(\text{alpha})$$

The squaring operator is applied to the logical triangular parameter, and the output of this operator is expressed in Equation (13). Equation (14) represents the final key (*K*) which is utilized for data authentication between the source and destination nodes, where *n* is the number of hours per day:

(13) s=sqrt(A−2m*(alpha))

(14) $$K=\sum_{i=1}^{n}\left(s_{i}+t_{i}+u\right)/2$$

Suppose *M* represents the original message from the source node to be encrypted and authenticated, *K* is the authentication key, *t* is the transmission time and *u* represent the unique sensor identification. The encryption for the TBSA takes in *M*, *K*, *t* and *u*, and generates the cipher-text *C* by using Equation (15) and it is expanded in Equation (16). At the same time, using *M*, TBSA generates the cipher-text *C* and a Tag of length *T_L_*. Moreover, this pair (*C*, *T_L_*) is transmitted to the receiver. The *M* can be variable, unlike other traditional symmetric ciphers where the data length needs to be fixed. This unique feature makes our proposed TBSA algorithm more energy-efficient. Because sometime the source node has less information than their block size of message, fixing this challenge is a mandatory requirement for many symmetric algorithms. Even for less information, fixed block sizes are sent in the traditional encryption algorithms. This wastes a lot of resources and power during data transmission between the source and destination nodes. Therefore, our proposed algorithm takes advantage of the variable block sizes for data encryption in order to provide energy-efficient security:

(15) C=(u⊕t)*M/(K)

(16) $$C=(M*(u⊕t))/(\sum_{t=1}^{n}\left(s+t+u\right)/2)$$

The receiver performs decryption on *C* to obtain *M* by using Equation (17) and it is expanded in Equation (18). Subsequently, the receiver guarantees that the received *Tag* is anticipated. If the destination node calculates dissimilar *Tag* then the cipher message will be unacceptable. In this case, if the *M* involves *n* blocks of data, then TBSA requires only *n* + 1 encryption to sustain both the confidentiality and integrity:

(17) M=C*K/(u⊕t)

(18) $$M=C*(\sum_{t=1}^{n}\left(s+t+u\right)/2)/(u⊕t)$$

The proposed TBSA, apart from data confidentiality, privacy, and integrity, also guarantees the data freshness by utilizing the incremental operator (*Io*) with limit up to *N*. The *Io* constantly gives an updated additional value similar to a counter, which is obtained from an incrementing operation. Consequently, it is very much essential that both the source and destination nodes utilize a distinct fresh nonce *N* for every transmission. Now, the proposed IoT-based home automation system includes the TBSA mechanism using the unique authentication key *K* and fresh nonce *N* for data encryption. Furthermore, the server obtains the periodic updates from the source node to check the confidentiality, privacy, integrity, and freshness of the collected information.

### 4.3. Security Analysis

This section demonstrates that the proposed IoT-based platform fulfills all the necessary security characteristics and requirements to oppose severe threats and attacks.

#### 4.3.1. Network Security Requirements Accomplishment

**Figure 6:**
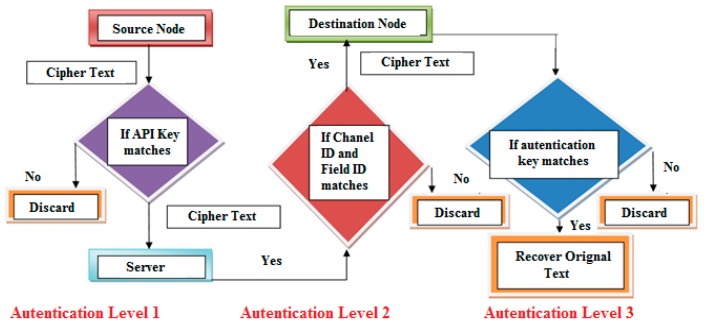
Three-levels of network security for IoT-based systems.

The IoT-based platform was implemented on the basis of the proposed security algorithm that provides network authentication at three different levels as shown in Figure 6. Authentication level 1 includes security of data transmitted from the source to the server, and if K_API_ does not match at the source node, communication will not be initiated. In our proposed system, the server validates the source node by verifying the onetime alias identity K_API_ and the track sequence number *Ts*, where only a valid sensor node can start communication with the server (authentication).

In smart home automation applications, the assessment of the source node location is very significant. In real-time applications, if there is no smart tracking method available, this can permit the hacker to transmit erroneous location by the initiating false signals. Our proposed IoT-based system can easily solve this problem. When the server desires to recognize the source node location, then it will exercise the tracking sequence number *Ts* identity which is the physical association between the sensor node and the server (secured localization). Consequently, the server will also inquire the node to present its identity. After that, the server substantiates the *Ts* provided by the node by contrasting it with the accumulated value of its record, and subsequently figures out the source node. Now, if the verification is successful, then the server can trust on the authenticity of the node (trustworthiness). Once the source entity identity is recognized, the source node cannot deny its authenticity for sending the originated messages thus fulfilling one of the major non-repudiation security requirements of IoT.

At level 2, the verification of channel ID and field ID are used to decide either to send data from the server to destination or not. Firstly, at this level of authentication, security service set identifier (SSID) and network password validation will be checked. After proper validation of the SSID and password, the server will check the channel and field IDs. The cipher text generated from source node using the proposed TBSA is transmitted to the server if there is a match in IDs. In cases where there is no match, the receiving node(s) will not be given access to the network (access control). To keep out potential attackers, it is needed to recognize each user and each device on the network so as to enforce security policies. After proper access control, all the network resources will be available in timely manner to the valid nodes (availability). Finally, at the receiver end, by using the authentication key, the original medical information can be recovered. Hence, WSN implemented based on the proposed TBSA provides a secured, less computationally complex, and energy-efficient data encryption to monitor data remotely from between the source and destination nodes.

#### 4.3.2. Data Security Requirements Accomplishment

The data security comprised of confidentiality, privacy, integrity, and data freshness. Since WSNs have a broadcast nature, so information could easily be changed and replayed by the attackers. Therefore, the proposed TBSA is used for data encryption; it assures all the requirements of data security, where any change in data or any replay endeavor by attacker can be accurately detected by means of a tag.

### 4.4. Energy Consumption Calculation

**Figure 7:**
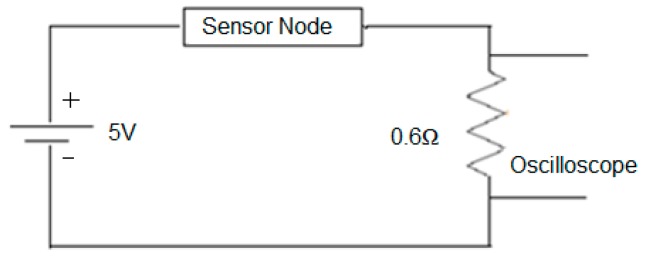
Experimental setup for investigating the energy consumption.

This study utilizes a circuit across the sensor node as shown in Figure 7, in order to evaluate the power utilization of the proposed TBSA in encrypting the original information. A digital oscilloscope (TDS 2024B, Tektronix, Beaverton, OR, USA) is utilized to compute the voltage (*V_s_*) across the resistor (0.6 ohms). Furthermore, Ohm’s law can be used to determine the current (*I_s_*) across the given resistor (*R*). In a series circuit, the current remains the same, so this current will be same across the resistor and sensor node. The power (*P_s_*) utilized by the node is determined by using Equation (18). Finally, the energy consumption (*E_s_*) of the sensor node will be computed by using Equation (19). The time taken by the proposed TBSA to encrypt the original text is represented by (*T_s_*):

(19) $$P_{s}=V_{s}*I_{s}$$

(20) $$E_{s}=P_{s}*T_{s}$$

## 5. Experiment Results and Discussion

This section mainly includes the temperature measurement using a TMP36 sensor (Sparkfun Electronics, Niwot, CO, USA), proposed TBSA-based data encryption to provide secure data transmission, and performance comparisons of proposed TBSA with traditional symmetric and hash ciphers, and security mechanisms developed for WSNs including Alarm-Net, PRESENT-GRP and PAWN in terms of energy-efficiency.

### 5.1. Temperature Measurement

In this study, low voltage temperature (TMP36) sensors were used to sense the environment temperature. The TMP36 utilizes a solid-state method to measure the temperature in °C, and it does not require any external calibration to deliver characteristic accuracies for different temperature levels. This sensor is operated by a single power supply having a range of 2.7 to 5.5 V. 

**Figure 8:**
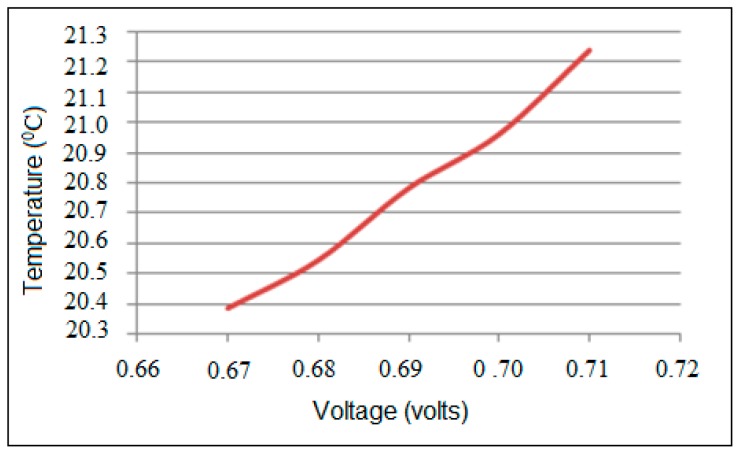
Temperature values against corresponding voltage values.

The actual output from TMP36 is in ADC; the ADC value should be converted into the correct voltage (*V_TMP_*). The ADC value (*ADC_output_*) is initially compared with the reference voltage of 5 V as shown in Equation (20) and then the characteristics of the TMP36 are used to obtain the temperature (*T*) as represented by the Equation (21). The linear relationship between voltage and temperature is shown in Figure 8:

(21) $$V_{TMP}=(ADC_{output}*5V)/1024$$

(22) $$T=(V_{TMP}-0.5V)/10$$

### 5.2. TBSA Encryption

The Intel Galileo-based source node collects the temperature values, further collected data is transmitted to the server by using Wi-Fi as a medium. Moreover, the original collected data is encrypted to cipher text by using the proposed TBSA prior to its transmission to the server in order to offer privacy and security to the original sensed information during transmission.

**Figure 9:**
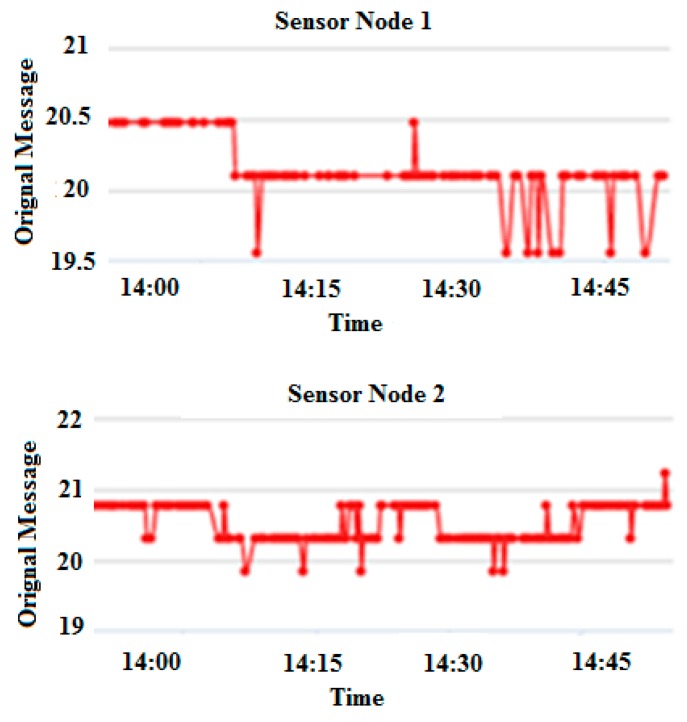
Original message from sensor node 1 and sensor node 2.

Consider a sensor node having unique ID (*u*) of 28, transmission (*t*) of 16 h, and *α* value of 44. The *u*, *s* and *t* represent the three sides of the triangle as shown in Figure 5. The value of the third side (*s*) is calculated to be 12.03 by using Equation (13). By inserting the values of *u*, *s* and *t* into Equation (14), a unique key with a value of 28.01 is generated. By inserting the unique key and the original message *M* (whose value is 21) into Equation (15), a cipher text with a value of 8.99 is obtained.In this study, two sensors nodes were used for performing the experiment. The unique IDs for sensor nodes 1 and 2 are 26 and 28, respectively. The experiment was performed between 13:30 and 15:30 on 24 May 2016. The original message obtained from both sensor nodes is shown in Figure 9. 

**Figure 10:**
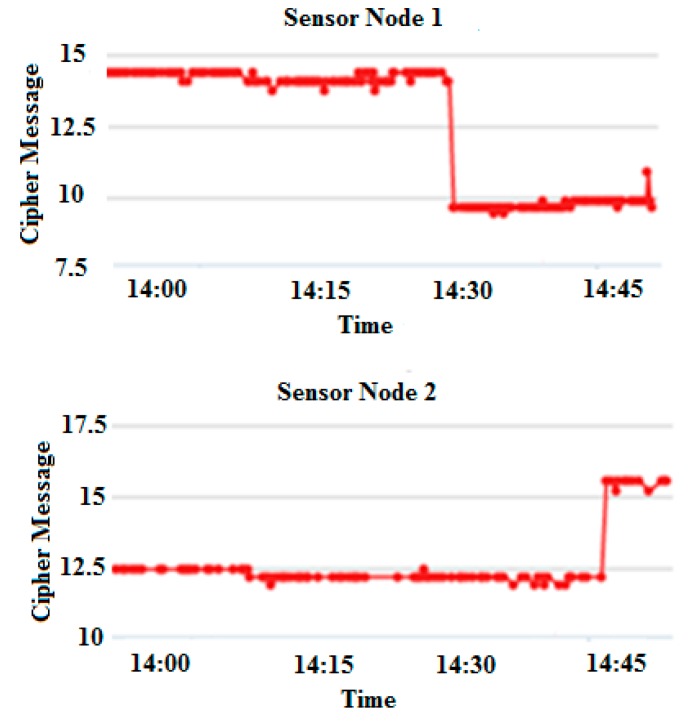
Cipher message from sensor node 1 and sensor node 2 by using proposed TBSA.

Further, the proposed TBSA is applied to the original messages from both sensor nodes in order to obtain the cipher messages as shown in Figure 10, which were later uploaded to the server. The received data at the server from a different node can be stored in different fields in the same channel or different fields in different channels. The different fields separate the information from each subject, through this way; data from each node can be understood easily. In this research, channel ID is 110980 for both sensor nodes. The single channel can have maximum support up to 8 different fields by using the ThingSpeak server. The field ID assigned for sensor node 1 is “1” and for a sensor node 2 is “5”.

### 5.3. Energy Consumption Comparison

The proposed security algorithm is based on a simple and efficient key generation procedure. Hence, it reduces the time requirement for key generation and encryption. As sensor nodes in WSNs are very tiny and have limited power, therefore, it is very significant to use less time-consuming security mechanism for WSNs. The energy consumption is linearly proportional to the processing time. That is, the higher taken to encrypt a message, the more the energy required. 

The TBSA consumes very less time for its implementation (7 µm). Complex key generation mechanisms are not used in this algorithm, that’s why TBSA consumes fewer resources for its data encryption. The power consumption by a sensor node during the implementation of TBSA algorithm is calculated by using the circuit as explained in Figure 7. The voltage (*Vs*) across the sensor nodes was 47 mA, which was calculated by using an oscilloscope. The resistance (*R*) of the resistor as determined by Ohm’s law is 0.6 Ω, the current (*Is*) was computed as 78.33 mA by substituting the values of *Vs* and *R* into Ohms law (*V = IR*), and the calculated power was calculated as 3.6 mW. The power consumed by the sensor nodes and the processing time for data encryption using the proposed TBSA were used to calculate the energy consumption across the nodes. After inserting 3.6 mW and 7-µm values into Equation (19), energy consumption per bit value of 0.025 Micro Joule was obtained. Since one byte has eight bits, therefore (8 × 0.025 = 0.2 Micro Joule/Byte) 0.2 Micro Joule/Byte will be required by the proposed TBSA algorithm to encrypt a byte of data.

**Figure 11:**
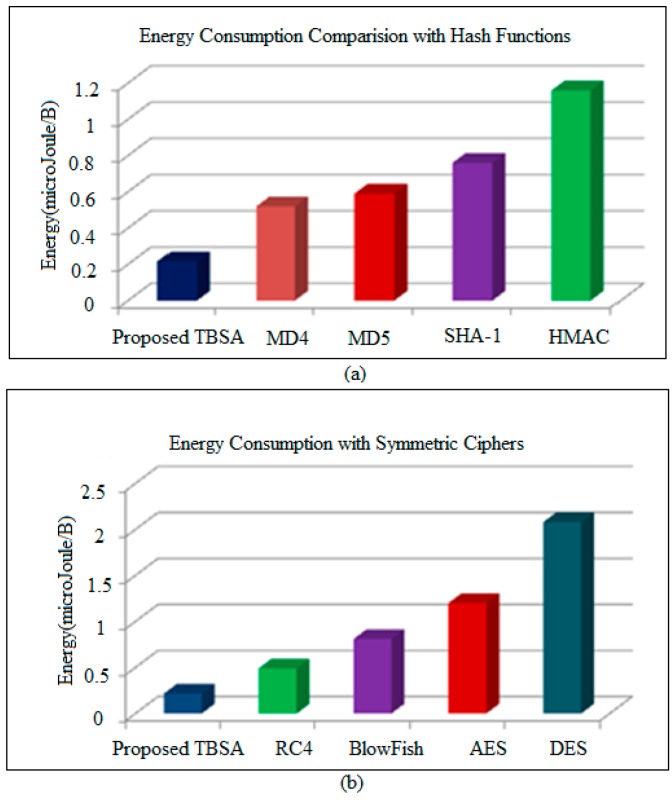
Energy consumption comparison of proposed TBSA (**a**) with Hash Functions; (**b**) with Symmetric Cipher.

In this research, the energy consumption by the TBSA algorithm is compared with the hash function and symmetric ciphers. The hash functions take a message of random size and produces an output of fixed-size value. A small change in the original text can affect the computation of a dissimilar hash value. They are mostly utilized for validating the reliability of data transmission between nodes. In this study, proposed TBSA is compared with the hash functions such as MD4, MD5 and SHA-1 in terms of energy-efficiency. The SHA1 is a new hash algorithm and has more steps for computation than MD4 and MD5. SHA1 is also considered to have better collision resistance than MD4 and MD5. This benefit of SHA-1 requires more energy than MD4 and MD5. The HMAC consumes more energy than MD4, MD5 and SHA-1. The HMAC is a keyed hash, and as the bit-length of the key is raised from 0 to 128 bits, the energy consumption fluctuates by a very minute amount. Figure 11a represents the energy consumption comparison of proposed TBSA algorithm with MD4, MD5, SHA-1 and HMAC. The energy consumed by TBSA, MD4, MD5, SHA-1 and HMAC is 0.20, 0.52, 0.59, 0.76 and 1.16 Micro Joule/Byte respectively, as shown in Table 1. 

Furthermore, this research includes the energy consumption comparisons of the proposed TBSA algorithm with symmetric ciphers. There are two major types of symmetric ciphers block ciphers and stream cipher to implement security. Block ciphers work on identical-length blocks of original text and cipher text. Examples of block ciphers comprise Data Encryption Standard (DES), Advanced Encryption Standard (AES), etc. The stream ciphers such as Rivest Cipher 4 (RC4) convert an original text to cipher text one bit (or byte) at a time. RC4 is considered as fast and efficient stream cipher, which is appropriate for encrypting information with more speed. However, it requires noteworthy encryption cost in comparison with other symmetric ciphers. Blowfish displays the largest difference between the energy consumption of key setup and encryption or decryption. The energy cost of key setup is the so high than encryption and decryption cost. It 64-bit cipher which executes encryption using straightforward processes and is intended to be efficient on 32-bit processors. This method is appropriate for applications where the secret key is not changing regularly (thereby allowing the significant overhead of key setup to be amortized by the low encryption cost). The design principle for AES algorithm is based on a combination of both substitution and permutation method. It is a variant of Rijndael which has a fixed block size of 128 bits, and a key size of 128, 192, or 256 bits. The DES is a symmetric cipher of the 64-bit block as it utilizes the identical key for both encryption and decryption. The key size for each round is 56 bits. Conversely, a 64-bit input is used for dissimilar keys generation. Figure 11b symbolizes the energy consumption comparison of TBSA algorithm with RC4, Blowfish, AES, and DES. The energy consumed by TBSA, RC4, Blowfish, AES and DES is 0.20, 0.49, 0.81, 1.2 and 2.08 Micro Joule/Byte, respectively, as expressed in Table 2.

**Figure 12:**
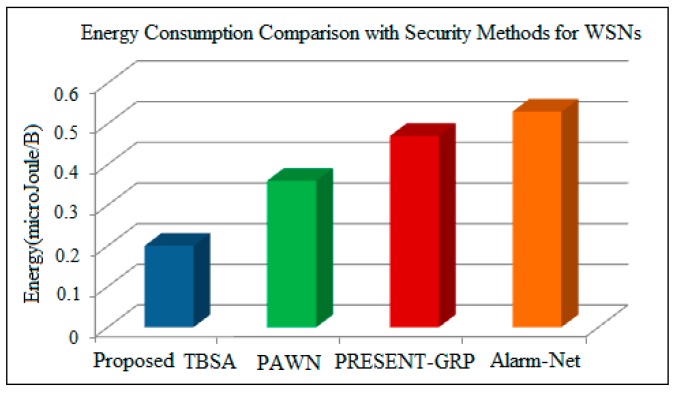
Energy consumption comparison of proposed TBSA with security mechanisms developed for WSNs.

Finally, security methods for WSN-based applications are compared with the proposed algorithm. The average data encryption times for the Alarm-Net [27], PRESENT-GRP [26], and PAWN [37] are 0.0123, 0.0156 and 0.01785 ms, respectively. Alarm-Net requires more processing cycles to generate unique keys, due to which more time is consumed for the encryption of original information than all compared methods. PAWN utilizes a simple procedure for cluster head assortment, so it needs a small processing time for data encryption than PRESENT-GRP and alarm-net. PRESENT-GRP uses a lightweight algorithm for implementing security, so it requires less processing time than Alarm-Net, but because of permutation boxes selection procedure a little more time is required for encryption than PAWN. The simple and efficient key generation mechanism are utilized in proposed TBSA (0.007 ms), therefore it demands less encryption time than Alarm-Net, PRESENT-GRP , and PAWN. The power consumption by sensor nodes for different approaches is 3.60, 3.66, 3.71 and 3.74 mW for PAWN, PRESENT-GRP, and Alarm-Net, respectively, as can be calculated by using the circuit shown in Figure 7. The energy consumption is the product of power and encryption time as shown in Equation (19) after inserting 3.6 mW and 0.007 ms value for the proposed TBSA in Equation (19), the energy consumption per bit is calculated as 0.025 Micro Joule. As one byte has eight bits, so 8 × 0.025 = 0.2 Micro Joule/Byte will be required by the TBSA algorithm to encrypt one byte of data. Hence, for PAWN, PRESENT-GRP, and Alarm-Net, the energy consumption can be calculated as 8 × 0.0123 × 3.66 = 0.36 Micro Joule/Byte, 8 × 0.0156 × 3.71 = 0.47 Micro Joule/Byte, and 8 × 0.01785 × 3.74 = 0.53 Micro Joule/Byte, respectively as shown in Table 3. The comparisons of proposed TBSA with PAWN, PRESENT-GRP, and Alarm-Net in terms of energy consumption are demonstrated in Figure 12.

It has been observed from Figure 11 and Figure 12 that the proposed TBSA algorithm requires less energy for its implementation in comparison with traditional security methods. As a simple and efficient key generation mechanism is used in TBSA, therefore less energy is consumed. Therefore, the proposed secure IoT-based home automation incorporates the proposed TBSA and low power Wi-Fi in WSNs with internet for providing efficient and secure data transmission among several nodes at wide coverage range.

## 6. Conclusions

This paper proposed secured IoT-based home automation applications using WSNs. In WSNs, because of the limited computational power of sensor nodes, an efficient security mechanism based on effective key generation mechanism which could accomplish all major data security requirements and consumes less processing time for data encryption is well needed. In this study a security algorithm, namely TBSA, based on a simple and efficient key generation procedure is developed. The proposed IoT integrates low power Wi-Fi and the proposed TBSA in WSNs with internet to provide additional benefits of increased coverage range and capability of supporting large number of sensor nodes due to usage of low power Wi-Fi module; it also consumes less processing time for data encryption because of the utilization of the proposed TBSA algorithm. The experimental results obtained from the hardware implementation have elaborated that the proposed algorithm TBSA is more energy-efficient for data encryption than all compared approaches. Furthermore, it has been verified in this study that developed IoT platform fulfills all major security requirements including network security (secure localization, non-repudiation, availability, access control, trustworthiness and authentication) and data security (confidentiality, privacy, integrity, and data freshness).

In near future, proposed IoT platform will be implemented for different applications such as medical monitoring and emergency response, agriculture, healthcare, energy management, and industrial automation. Additionally, we will develop an efficient biometric-based security algorithm based on Heart Rate Variability (HRV) to secure modern healthcare system using Wireless Body Sensor Networks (WBSNs). The time-domain parameters of HRV such as Standard Deviation of NN interval (SDNN) and Root-Mean Squared of the Successive Differences (RMSSD) along with TBSA will be used for key generation or entity identifications in WBSNs. 

## Figures and Tables

**Table 1:** Energy Consumption Comparison with Hash Functions.

S. NO	Technique/Method	Energy Consumption (Micro Joule/Byte)
1	Proposed TBSA	0.20
2	MD4	0.52
3	MD5	0.59
4	SHA-1	0.76
5	HMAC	1.16

TBSA: Triangle Based Security Algorithm, MD4: Message Digest 4, MD5: Message Digest 5, SHA-1: Secure Hash Algorithm 1, HMAC: Hash Message Authentication Code.

**Table 2:** Energy consumption comparison with symmetric cipher.

S. NO	Technique/Method	Energy Consumption (Micro Joule/Byte)
1	Proposed TBSA	0.20
2	RC4	0.49
3	Blowfish	0.81
4	AES	1.20
5	DES	2.80

TBSA: Triangle Based Security Algorithm, RC4: Rivest Cipher 4, AES: Encryption Standard, DES: Advanced Encryption Standard

**Table 3:** Energy consumption comparison with security methods designed for WSNs.

S. NO	Technique/Method	Energy Consumption (Micro Joule/Byte)
1	Proposed TBSA	0.20
2	PAWN	0.36
3	PRESENT-GRP	0.47
4	Alarm-Net	0.53
