# Erratum: Sandeep P., et al*. *A Novel Secure IoT-Based Smart Home Automation System Using a Wireless Sensor Network. *Sensors *2017, *17*, 69

**DOI:** 10.3390/s17030606

**Published:** 2017-03-16

**Authors:** Sandeep Pirbhulal, Heye Zhang, Md. Eshrat E Alahi, Hemant Ghayvat, Subhas Chandra Mukhopadhyay, Yuan-Ting Zhang, Wanqing Wu

**Affiliations:** 1 Shenzhen Institutes of Advanced Technology, Chinese Academy of Sciences, Shenzhen 518055, China; sandeep@siat.ac.cn (S.P.); hy.zhang@siat.ac.cn (H.Z.); 2 Research Center for Biomedical Information Technology, Shenzhen Institutes of Advanced Technology, 1068 Xueyuan Avenue, Shenzhen University Town, Shenzhen 518055, China; 3 Shenzhen College of Advanced Technology, University of Chinese Academy of Sciences, Shenzhen 518055, China; 4 School of Engineering and Advanced Technology, Massey University, Palmerston North 4442, New Zealand; ealahi@yahoo.com (M.E.E.A.); ghayvat@gmail.com (H.G.); 5 Department of Engineering, Macquarie University, Sydney 2109, Australia; 6 Joint Research Centre for Biomedical Engineering, Chinese University of Hong Kong, Shatin N.T., Hong Kong, China; ytzhangapple@icloud.com

The authors wish to make the following corrections to their paper [1]:

The first affiliation of authors was incorrect in our published paper in Sensors [1]. Therefore, it is corrected from “Institute of Biomedical and Health Engineering, Shenzhen Institutes of Advanced Technology, Shenzhen 518055, China” to “Shenzhen Institutes of Advanced Technology, Chinese Academy of Sciences, Shenzhen 518055, China”.

The authors would like to apologize for any inconvenience caused to the readers by this change. The manuscript will be updated and the original will remain online on the article webpage.

## Supplementary Materials

Supplementary File 1
