# Supplementary material for: Erratum: Sandeep P., et al. A Novel Secure IoT-Based Smart Home Automation System Using a Wireless Sensor Network. Sensors 2017, 17, 69
Source: Sensors (Basel). 2017 Mar 16;17(3):606. doi: 10.3390/s17030606 (PMC5375892; doi:10.3390/s17030606)
Supplement: Supplementary File 1 [file sensors-17-00606-s001.zip › sensors-17-00069-tex-v1.zip!/sensors-17-00069-g001.pdf]

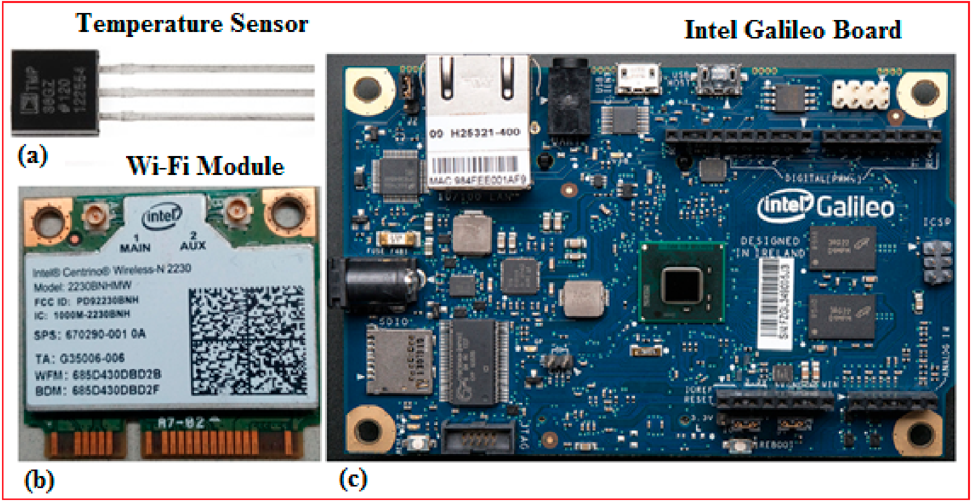
Temperature Sensor
(a)
Wi-Fi Module
1 MAIN
2 AUX
Intel® Centrino® Wireless-N 2230
Model: 2230BNHMW
FCC ID: PD92230BNH
IC: 1000M-2230BNH
SPS: 670290-001 0A
TA: G35006-006
WFM: 685D430DBD2B
BDM: 685D430DBD2F
(b)
Intel Galileo Board
intel Galileo
DESIGNED IN IRELAND
(c)
