# Supplementary material for: Erratum: Sandeep P., et al. A Novel Secure IoT-Based Smart Home Automation System Using a Wireless Sensor Network. Sensors 2017, 17, 69
Source: Sensors (Basel). 2017 Mar 16;17(3):606. doi: 10.3390/s17030606 (PMC5375892; doi:10.3390/s17030606)
Supplement: Supplementary File 1 [file sensors-17-00606-s001.zip › sensors-17-00069-tex-v1.zip!/sensors-17-00069-g002.pdf]

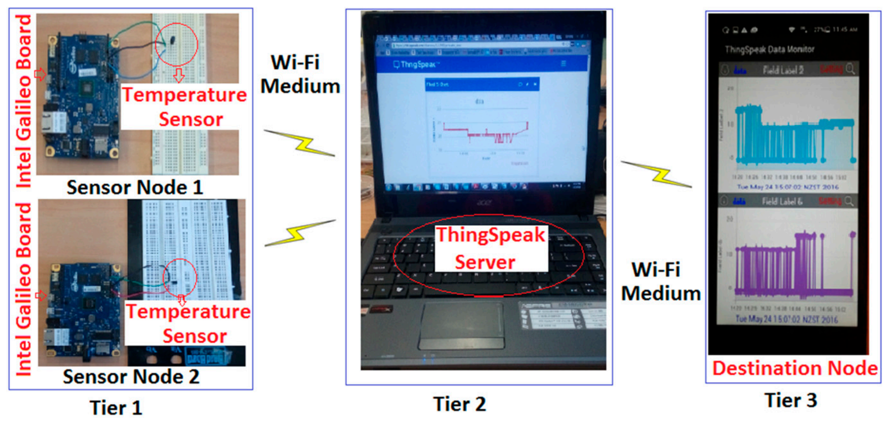

Intel Galileo Board
Temperature Sensor
Sensor Node 1
Intel Galileo Board
Temperature Sensor
Sensor Node 2
Tier 1
Wi-Fi Medium
ThingSpeak Server
Tier 2
Wi-Fi Medium
ThingSpeak Data Monitor
Tue May 24 15:07:02 NZST 2016
Tue May 24 15:07:02 NZST 2016
Destination Node
Tier 3
