# Supplementary material for: Erratum: Sandeep P., et al. A Novel Secure IoT-Based Smart Home Automation System Using a Wireless Sensor Network. Sensors 2017, 17, 69
Source: Sensors (Basel). 2017 Mar 16;17(3):606. doi: 10.3390/s17030606 (PMC5375892; doi:10.3390/s17030606)
Supplement: Supplementary File 1 [file sensors-17-00606-s001.zip › sensors-17-00069-tex-v1.zip!/sensors-17-00069-g003.pdf]

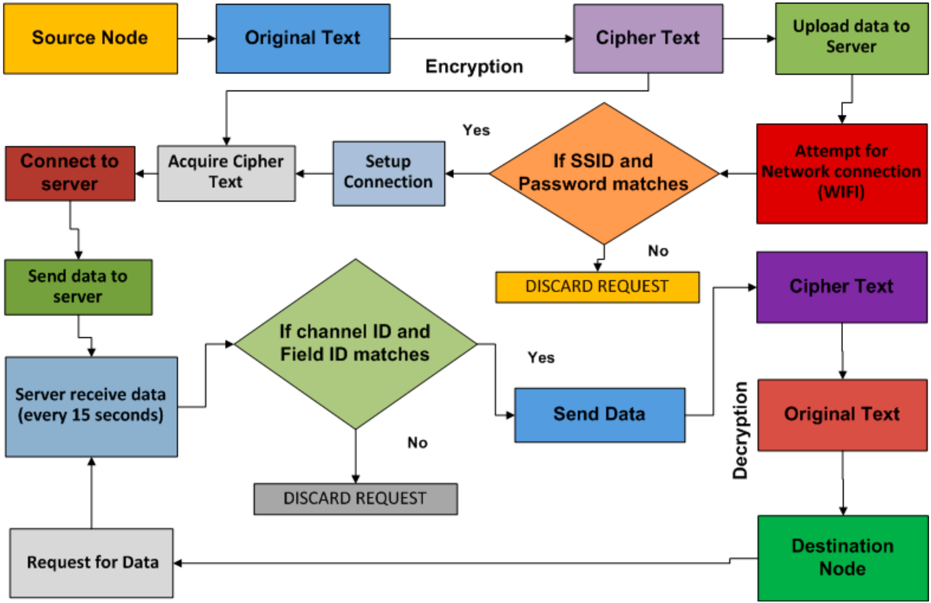
Source Node
Original Text
Encryption
Cipher Text
Upload data to Server
Attempt for Network connection (WIFI)
If SSID and Password matches
Yes
No
DISCARD REQUEST
Setup Connection
Acquire Cipher Text
Connect to server
Send data to server
Server receive data (every 15 seconds)
If channel ID and Field ID matches
Yes
No
DISCARD REQUEST
Send Data
Cipher Text
Decryption
Original Text
Destination Node
Request for Data
