# Supplementary material for: Erratum: Sandeep P., et al. A Novel Secure IoT-Based Smart Home Automation System Using a Wireless Sensor Network. Sensors 2017, 17, 69
Source: Sensors (Basel). 2017 Mar 16;17(3):606. doi: 10.3390/s17030606 (PMC5375892; doi:10.3390/s17030606)
Supplement: Supplementary File 1 [file sensors-17-00606-s001.zip › sensors-17-00069-tex-v1.zip!/sensors-17-00069-g006.pdf]

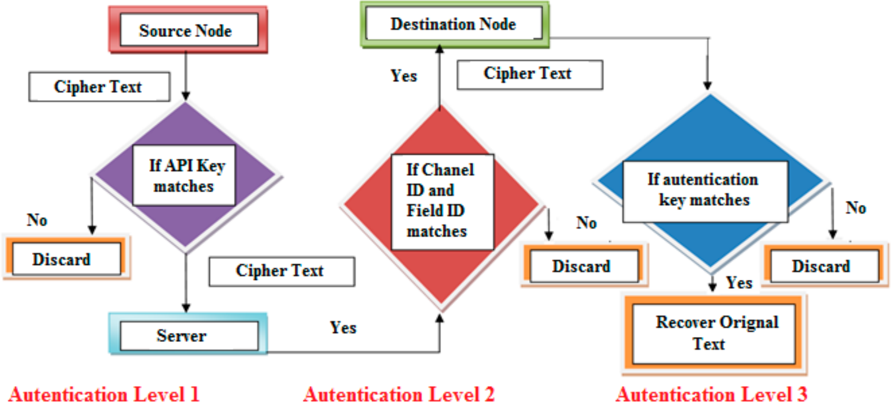
Source Node
Cipher Text
If API Key matches
No
Discard
Cipher Text
Server
Yes
Destination Node
Yes
Cipher Text
If Chanel ID and Field ID matches
No
Discard
If autentication key matches
No
Discard
Yes
Recover Orignal Text
Autentication Level 1
Autentication Level 2
Autentication Level 3
