# Supplementary material for: Erratum: Sandeep P., et al. A Novel Secure IoT-Based Smart Home Automation System Using a Wireless Sensor Network. Sensors 2017, 17, 69
Source: Sensors (Basel). 2017 Mar 16;17(3):606. doi: 10.3390/s17030606 (PMC5375892; doi:10.3390/s17030606)
Supplement: Supplementary File 1 [file sensors-17-00606-s001.zip › sensors-17-00069-tex-v1.zip!/sensors-17-00069-g008.pdf]

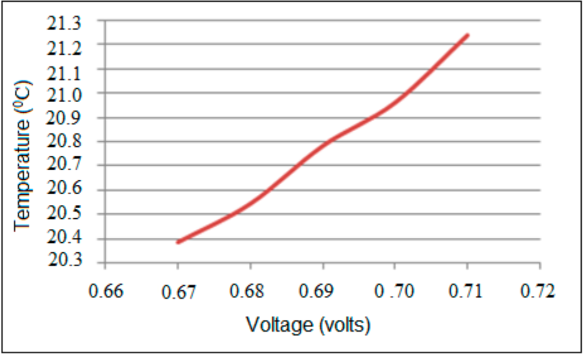

21.3
21.2
21.1
21.0
20.9
20.8
20.7
20.6
20.5
20.4
20.3
Temperature (^{0}C)
0.66
0.67
0.68
0.69
0 .70
0.71
0.72
Voltage (volts)
