# Supplementary material for: Erratum: Sandeep P., et al. A Novel Secure IoT-Based Smart Home Automation System Using a Wireless Sensor Network. Sensors 2017, 17, 69
Source: Sensors (Basel). 2017 Mar 16;17(3):606. doi: 10.3390/s17030606 (PMC5375892; doi:10.3390/s17030606)
Supplement: Supplementary File 1 [file sensors-17-00606-s001.zip › sensors-17-00069-tex-v1.zip!/sensors-17-00069-g011.pdf]

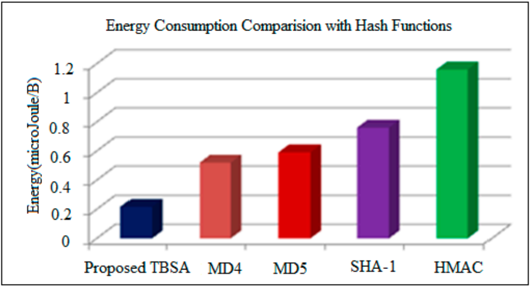

Energy Consumption Comparision with Hash Functions
Energy(microJoule/B)
1.2
1
0.8
0.6
0.4
0.2
0
Proposed TBSA
MD4
MD5
SHA-1
HMAC

(a)

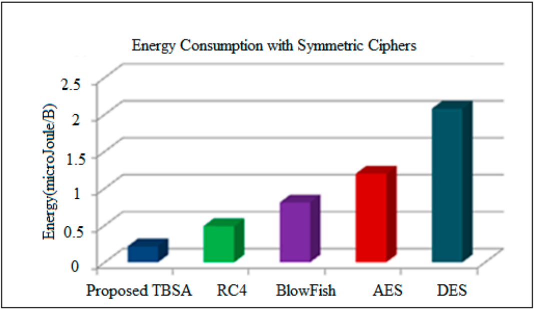

Energy Consumption with Symmetric Ciphers
Energy(microJoule/B)
2.5
2
1.5
1
0.5
0
Proposed TBSA
RC4
BlowFish
AES
DES

(b)
